# Laparoscopic repair of a traumatic diaphragmatic hernia with repeated colon incarcerations 7 years after injury: a case report

**DOI:** 10.1186/s40792-023-01791-9

**Published:** 2023-12-11

**Authors:** Satoshi Higuchi, Tsuyoshi Takahashi, Yukinori Kurokawa, Takuro Saito, Kazuyoshi Yamamoto, Kota Momose, Kotaro Yamashita, Koji Tanaka, Tomoki Makino, Kiyokazu Nakajima, Hidetoshi Eguchi, Yuichiro Doki

**Affiliations:** Department of Gastroenterological Surgery, Osaka University Graduate School of Medicine, 2-2 Yamadaoka, Suita, Osaka 565-0871 Japan

**Keywords:** Colon incarceration, Laparoscopic surgery, Traumatic diaphragmatic hernia

## Abstract

**Background:**

A diaphragmatic hernia is a prolapse of the abdominal organs into the thoracic cavity through a hole in the diaphragm. Traumatic diaphragmatic injuries are rare and usually occur after blunt or penetrating thoracic or abdominal traumas. Blunt diaphragmatic rupture rarely accounts for immediate mortality and may go clinically silent until complications occur which can be life threatening. It usually present late with intrathoracic herniation of abdominal viscera and carry a high mortality rate. We experienced a very rare case who showed repeated colon incarcerations 7 years after injury. And, we operated laparoscopically.

**Case presentation:**

A 64-year-old man presented with multiple left rib fractures that occurred during an accident. After 7 years, he visited the emergency department with the chief complaint of left shoulder pain and epicardial pain after eating. He was diagnosed with transverse colon incarceration due to a left diaphragmatic hernia by computed tomography (CT) and X-ray imaging. Surgical repair was recommended, but he refused as the symptoms improved. Fourteen months later, the patient revisited the hospital in similar symptoms and improved spontaneously. He consulted our hospital for the surgical indication. We recommended that he undergo surgery, showing images of the X-ray and CT when his transverse colon was obstructed and he felt pain and when symptoms improved. Finally, he decided to undergo surgery. We performed diaphragmatic hernia repair with laparoscopic direct suturing in good view. The patient experienced an uneventful postoperative recovery period. The absence of diaphragmatic herniation recurrence was confirmed seven months after surgery.

**Conclusions:**

We experienced a traumatic diaphragmatic hernia with repeated colon incarcerations 7 years after injury and performed surgical repair laparoscopically.

**Supplementary Information:**

The online version contains supplementary material available at 10.1186/s40792-023-01791-9.

## Background

A diaphragmatic hernia is a prolapse of the abdominal organs into the thoracic cavity through a hole in the diaphragm, occurring congenitally in newborns or acquired secondarily to trauma. Traumatic diaphragmatic injuries are rare and usually occur after blunt thoracic, abdominal (3–5%), or penetrating (3–15%) trauma. In blunt trauma, 65–85% of cases are left-sided and 15–35% right-sided [1], as the right diaphragm is usually protected by the liver [2].

More than 30% of diaphragmatic hernia cases are identified late, with onset varying from 24 h to 50 years after injury [3]. Symptoms vary widely depending on hernia location and the organs involved, making diagnosis difficult. Once diagnosed, diaphragmatic hernia should be repaired as soon as possible to reduce the risk of subsequent complications [4].

Here, we performed laparoscopic repair for a left-sided traumatic diaphragmatic hernia with persistent 7 years after injury.

## Case presentation

A 64-year-old man presented with multiple fractures of the 8th to 12th left ribs after an accident in December 2014. The patient was treated conservatively, with no ongoing symptoms noted. In 2019 and 2020, he had been seen in hospital with the main complaint of left shoulder pain, which was under observation. In March 2021, he visited the emergency department complaining of left shoulder pain and epicardial pain after eating. A left diaphragmatic hernia was diagnosed by computed tomography (CT) and radiography, and surgical repair was recommended. However, the patient refused as the symptoms improved. Fourteen months later, the patient revisited the hospital in similar circumstances and improved spontaneously. He consulted our hospital for the surgical indication. We recommended that he undergo surgery, showing images of the X-ray and CT when his transverse colon was obstructed and he felt pain and when symptoms improved. Finally, he decided to undergo surgery.

**Fig. 1:**
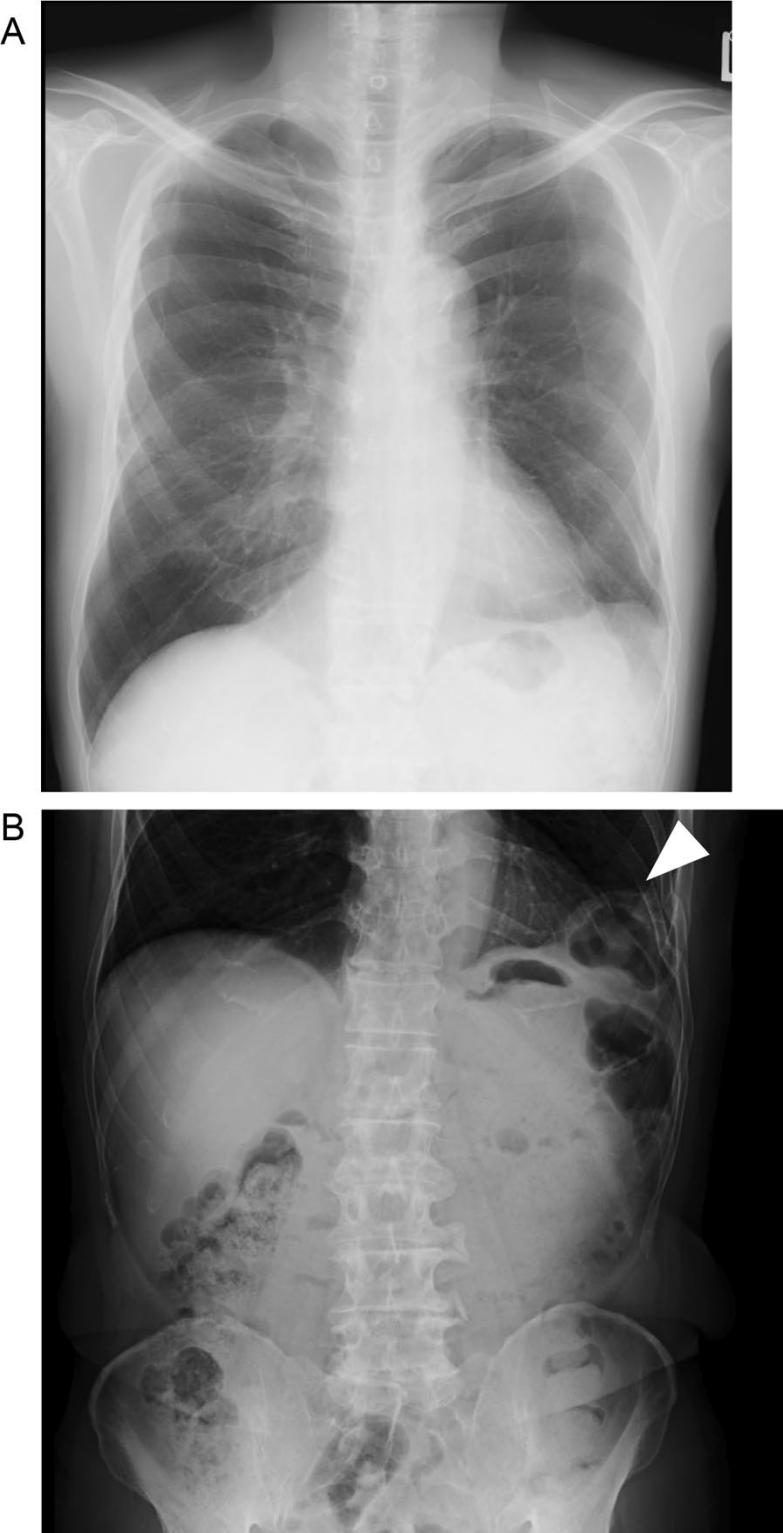
Preoperative radiographs. Chest radiographs show intra-abdominal tissue evacuated into the left thoracic cavity in the absence of pain (**A**) and presence of pain (**B**). We confirmed the colon incarceration (white arrowhead)

**Fig. 2:**
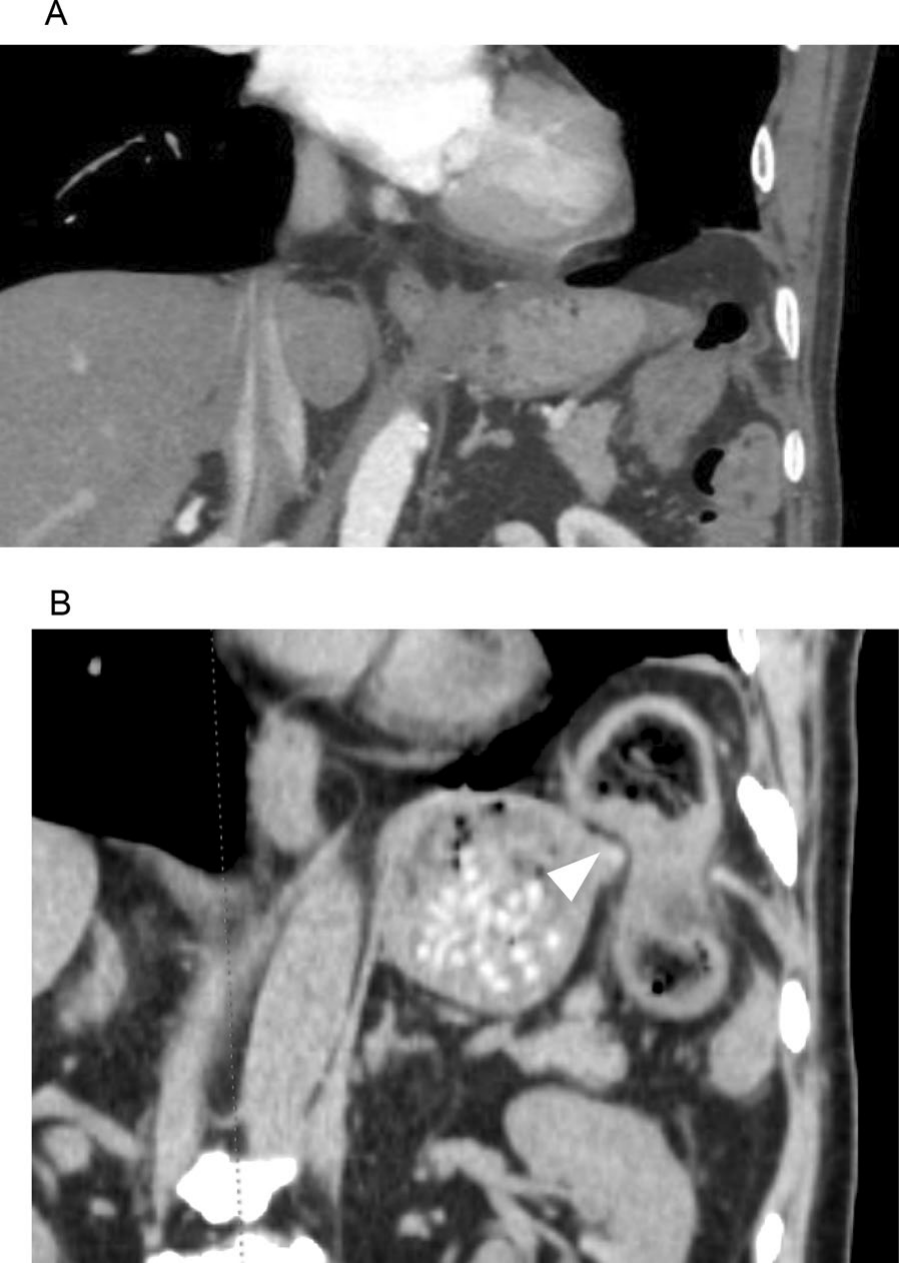
Preoperative CT images. CT images show intra-abdominal tissue evacuated into the thoracic cavity in the absence of pain (**A**) and presence of pain (**B**). We confirmed the colon incarceration (white arrowhead)

The chest radiograph in the absence of symptoms showed prolapse of intra-abdominal tissue into thoracic cavity (Fig. 1A). In the presence of symptoms, it revealed intestinal air in the left lower lung field (Fig. 1B). CT images in the absence and presence of symptoms are shown in Fig. 2. It shows intra-abdominal tissue evacuated into the thoracic cavity, even in the absence of pain. When the patient felt pain, the transverse colon was obstructed where it passed through the diaphragm into the thoracic cavity. Because the symptoms occurred frequently, he decided to receive the operation.

**Fig. 3:**
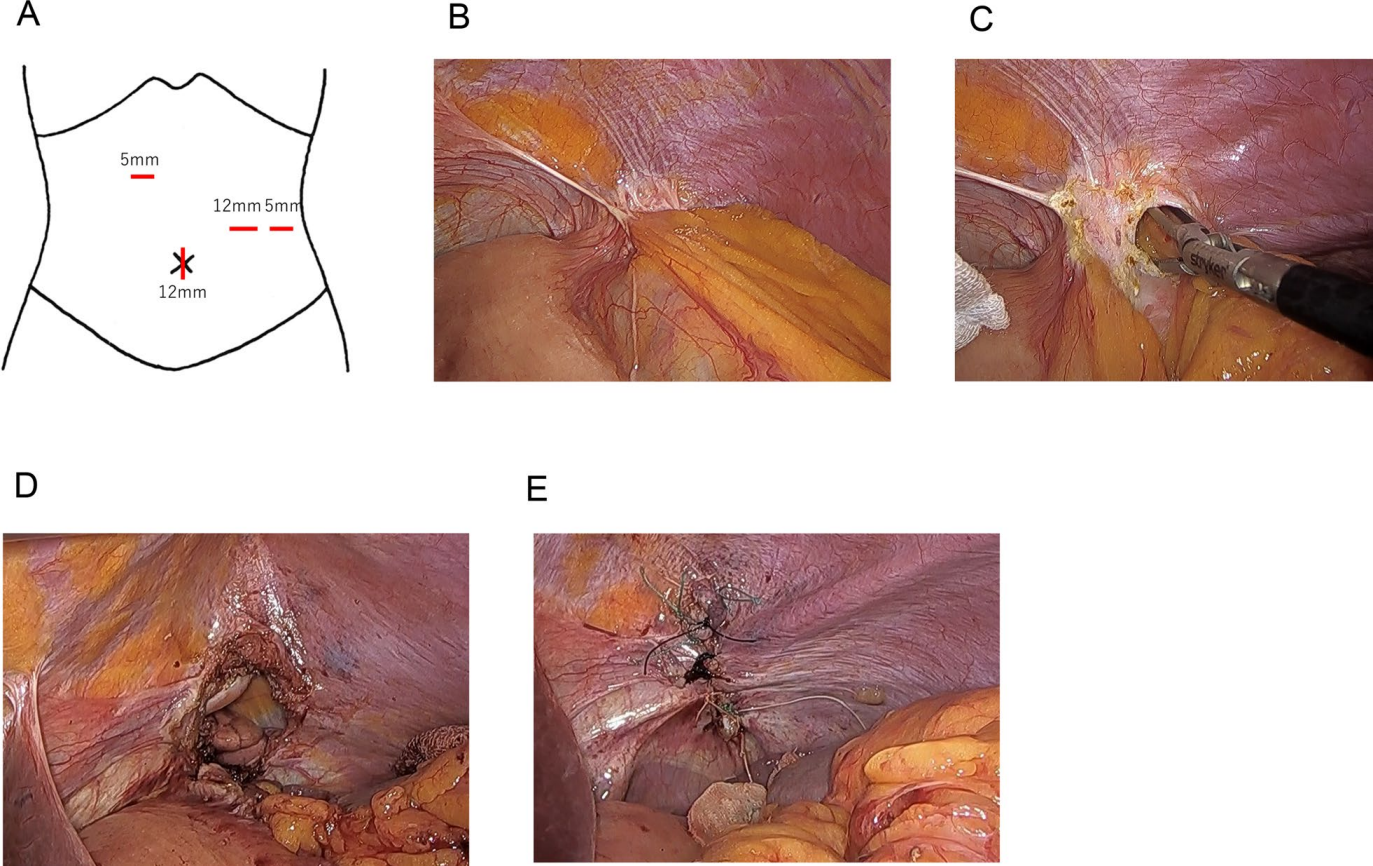
Operative findings. Ports placement (**A**). We confirmed colon incarceration laparoscopically, dissected around the hernial orifice, and pulled the prolapsed transverse colon and omentum back into the abdominal cavity (**B**, **C**). The size of the hernia ring was 5 × 3 cm (**D**). We closed with single nodal sutures using non-absorbable sutures and did not use a mesh (**E**)

The patient position was right semi-supine position. We inserted a 12 mm port through the umbilicus, a 5 mm port through the right side of the abdomen, and 12 mm and 5 mm ports through the left side of the abdomen, respectively (Fig. 3A) and started laparoscopically. We confirmed colon incarceration (Fig. 3B). We dissected around the hernial orifice, and pulled the prolapsed transverse colon and omentum back into the abdominal cavity (Fig. 3C). The size of the hernia ring was 5 × 3 cm (Fig. 3D). We closed with single nodal sutures using non-absorbable sutures and did not use a mesh (Fig. 3E). The operating time was 174 min and loss of blood was small amount.

**Fig. 4:**
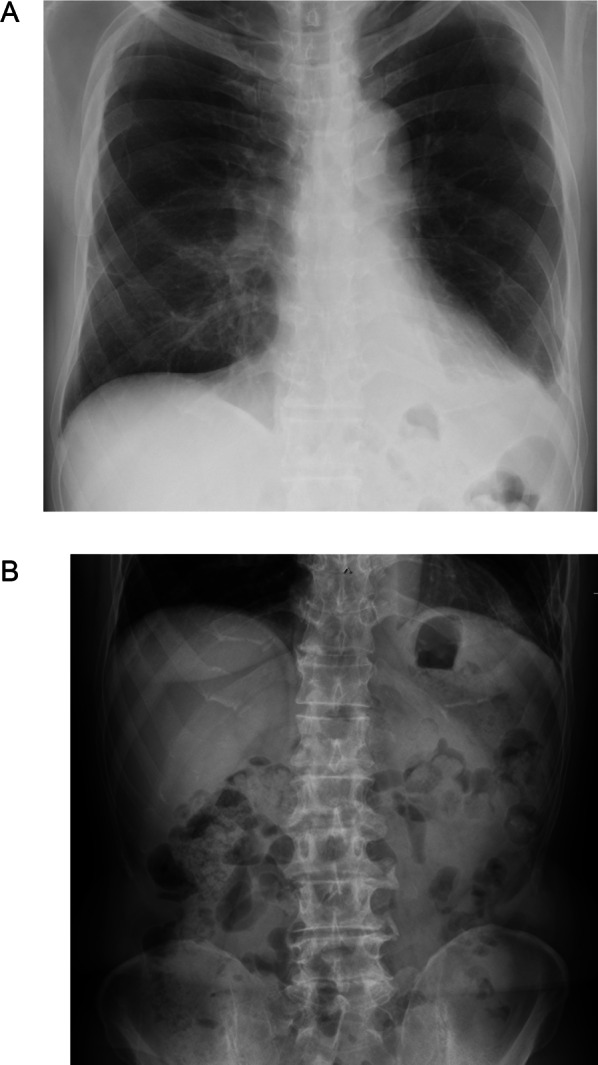
Postoperative radiographs. Chest (**A**) and abdominal (**B**) radiographs do not show prolapse of intra-abdominal tissue into thoracic cavity

The patient’s postoperative course was good. The patient started eating on POD 2 and was discharged at POD 8. The absence of diaphragmatic herniation recurrence was confirmed seven months after surgery (Fig. 4).

## Discussion

The diagnosis of a traumatic diaphragmatic hernia (TDH) can be challenging as the physical examination may be unremarkable; however, a high index of suspicion is important to ensure early diagnosis. TDH is incorrectly diagnosed in up to 33% of cases during the immediate post-traumatic period. If left untreated, mortality rates are between 25 and 80% in patients with strangulation of the incarcerated viscera [1]. TDH can be diagnosed by chest radiography, but it is difficult to recognize small lesions, and abnormal shadows due to pleural effusion may limit visibility. CT can show the location and size of the damaged diaphragm and prolapsed organ, thus improving the accuracy of diagnosis. In this case, because the patient showed symptoms approximately seven years after the injury, diagnosis was difficult. However, coronal CT images can easily reveal a prolapsed colon.

TDH is reported to occur more often on the left side as the right diaphragm is usually protected by the liver [2]. In the 36 traumatic diaphragmatic hernias reported in the past 5 years (Additional file 1), 34 cases were left-sided cases. And in the 9 cases in which details of the diaphragmatic defect were given in the text or were known from intraoperative photographs, 8 cases (88.9%) were centrally. It may be that the central area may be anatomically the weakest by the trauma.

According to Grimes, there are three phases of diaphragmatic hernia [5]. The acute phase occurs when the diaphragm is injured. In the delayed phase, the organs transiently prolapse, often with intermittent or no specific symptoms. The obstruction phase involves complications of long-standing herniation, manifesting as obstruction, strangulation, and rupture [5]. Therefore, once diagnosed, patients should be treated as soon as possible. Almost all cases previously reported were operated emergently after the appearance of acute serious symptoms. In this case, it took approximately one year from diagnosis to treatment. Although intermittent pain was observed, the images showed that the transverse colon was constantly prolapsing. Thus the patient was considered to be in the obstruction phase; fortunately, the bowel was not strangulated. As mentioned above, the patient was in the obstructive phase and the trapped transverse colon should not be released. Despite this, the obstruction was getting worse and better spontaneously. By the operative findings, we speculated that the colon was incompletely prolapsed through the narrow hernial orifice, and the symptoms developed by some kind of trigger that caused obstruction to passage, which was unusual and characteristic in this case.

TDH can be repaired by laparotomy, laparoscopy, or thoracoscopy, depending on the experience and skills of the surgeon [1]. Complete reduction of the herniated organs back into the abdomen and watertight closure of the diaphragm will help avoid recurrence [4]. The laparoscopic approach is superior in several respects, including allowing visualization of the entire abdominal cavity and lesions. Furthermore, we can find the adverse events such as hemorrhage, organ damage, and intestinal necrosis, not to mention smaller wounds and faster postoperative recovery. Fixing the mesh to the abdominal side of the diaphragm is easier than fixing it to the thoracic side because there is more working space in the abdominal cavity [2]. In contrast, in cases with adhesions between the prolapsed organ and the thoracic organ (e.g., lung or pericardium), a thoracoscopic approach is easier.

There have been several reports on the use of meshes. Shao et al. reported the utility of American Association for the Surgery of Trauma (AAST) classification, where diaphragmatic injuries are clustered into five grades: I (contusion), II (laceration ≤ 2 cm), III (laceration 2–10 cm), IV (laceration ˃10 cm with tissue loss ≤ 25 cm^2^), and V (laceration with tissue loss ˃25 cm^2^) [6]. They reported that defects larger than 20–30 cm^2^ (grades IV and V of the AAST Classification) usually require prosthesis reinforcement [7]. According to Liu et al., a hernia ring with a diameter of < 5 cm can be directly sutured and repaired. Where the hernia ring is ≥ 5 cm, an anti-adhesion mesh of appropriate size was fixed at a position approximately 3–5 cm beyond the edge of the hernia ring [2]. Biological mesh is preferentially used as it reduces adhesion formation, improves biocompatibility, decreases inflammatory response, optimizes neovascularization, lowers rates of hernia recurrence, increases resistance to infections, and lowers the risk of displacement [1]. Non-absorbable simple sutures are preferred over absorbable sutures in terms of recurrence [4]. In our case, because the hernia was not so large (approximately 15 cm^2^), we decided to close with single nodal sutures without a mesh.

In conclusion, we experienced a very rare case who showed repeated colon incarcerations 7 years after injury. We were able to perform a laparoscopic procedure to repair a TDH with a good field of vision.

### Supplementary Information

**Additional file 1.** References.

## Data Availability

The data supporting the findings of this study are available from the corresponding author upon reasonable request.

## Abbreviations

CT: Computed tomography
TDH: Traumatic diaphragmatic hernia
AAST: American Association for the Surgery of Trauma
