# Supplementary material for: Laparoscopic repair of a traumatic diaphragmatic hernia with repeated colon incarcerations 7 years after injury: a case report
Source: Surg Case Rep. 2023 Dec 11;9:212. doi: 10.1186/s40792-023-01791-9 (PMC10710970; doi:10.1186/s40792-023-01791-9)
Supplement: Supplementary file 1 — Additional file 1. References. [file 40792_2023_1791_MOESM1_ESM.docx]

**Additional file 1**

**Reference****s**

1. Abu Halimah, J., Laparoscopic management of large bowel obstruction caused by late post-traumatic diaphragmatic hernia: A case report. Int J Surg Case Rep, 2023. **111**: p. 108816.

2. Charara, R.H., et al., Laparoscopic Repair of Acute Traumatic Diaphragmatic Hernia: A Case Report. Cureus, 2023. **15**(6): p. e40959.

3. Choi, J., et al., LAPRA-TY for laparoscopic repair of traumatic diaphragmatic hernia without intracorporeal knot tying. Trauma Surg Acute Care Open, 2019. **4**(1): p. e000334.

4. Elkbuli, A., et al., Traumatic diaphragmatic rupture successfully managed in 4-year-old patient: Case report and literature review. Int J Surg Case Rep, 2020. **72**: p. 237-240.

5. Jain, N., et al., Delayed presentation of a post-traumatic large right diaphragmatic hernia displacing liver and gallbladder - A case report. Asian J Endosc Surg, 2022. **15**(2): p. 388-392.

6. Kori, M., et al., Laparoscopic repair and total gastrectomy for delayed traumatic diaphragmatic hernia complicated by intrathoracic gastric perforation with tension empyema: a case report. Surg Case Rep, 2022. **8**(1): p. 117.

7. Kumar, A., et al., Thoracolaparoscopic repair of diaphragmatic hernias. Indian J Thorac Cardiovasc Surg, 2021. **37**(5): p. 558-564.

8. Liu, Q., et al., Treatment of Chronic Traumatic Diaphragmatic Hernia Based on Laparoscopic Repair: Experiences From 23 Cases. Front Surg, 2021. **8**: p. 706824.

9. Nishikawa, S., et al., Laparoscopic repair of traumatic diaphragmatic hernia with colon incarceration: A case report. Asian J Endosc Surg, 2021. **14**(2): p. 258-261.

10. Shichiri, K., et al., Minimally invasive repair of right-sided blunt traumatic diaphragmatic injury. BMJ Case Rep, 2020. **13**(11).

11. Singh, D., S. Aggarwal, and S. Vyas, Laparoscopic repair of recurrent traumatic diaphragmatic hernia. J Minim Access Surg, 2020. **16**(2): p. 166-168.

12. Tebha, S.S., et al., Angiotensin converting enzyme inhibitor associated spontaneous herniation of liver mimicking a pleural mass: A case report. World J Hepatol, 2022. **14**(4): p. 854-859.

13. Toh, P.Y., S. Parys, and Y. Watanabe, Traumatic diaphragmatic rupture: delayed presentation following a SCUBA dive. BMJ Case Rep, 2020. **13**(9).

14. Yoshimine, S., et al., Laparoscopic-assisted thoracoscopic repair of latent traumatic diaphragmatic hernia: A case report. Asian J Endosc Surg, 2023. **16**(4): p. 800-803.
